# Lipoproteins Contribute to the Anti-inflammatory Capacity of *Lactobacillus plantarum* WCFS1

**DOI:** 10.3389/fmicb.2020.01822

**Published:** 2020-07-29

**Authors:** I-Chiao Lee, Iris I. van Swam, Sjef Boeren, Jacques Vervoort, Marjolein Meijerink, Nico Taverne, Marjo Starrenburg, Peter A. Bron, Michiel Kleerebezem

**Affiliations:** ^1^Host-Microbe Interactomics Group, Wageningen University & Research, Wageningen, Netherlands; ^2^TiFN Food & Nutrition, Wageningen, Netherlands; ^3^NIZO Food Research, Ede, Netherlands; ^4^Laboratory of Biochemistry, Wageningen University & Research, Wageningen, Netherlands

**Keywords:** *Lactobacillus*, lipoproteins, human immune system, proteomics, prolipoprotein diacylglyceryl transferase, LGT, *Lactobacillus plantarum*, probiotics

## Abstract

Bacterial lipoproteins are well-recognized microorganism-associated molecular patterns, which interact with Toll-like receptor (TLR) 2, an important pattern recognition receptor of the host innate immune system. Lipoproteins are conjugated with two- or three-acyl chains (di- or tri-acyl), which is essential for appropriate anchoring in the cell membrane as well as for the interaction with TLR2. Lipoproteins have mostly been studied in pathogens and have established roles in various biological processes, such as nutrient import, cell wall cross-linking and remodeling, and host-cell interaction. By contrast, information on the role of lipoproteins in the physiology and host interaction of probiotic bacteria is scarce. By deletion of *lgt*, encoding prolipoprotein diacylglyceryl transferase, responsible for lipidation of lipoprotein precursors, we investigated the roles of the collective group of lipoproteins in the physiology of the probiotic model strain *Lactobacillus plantarum* WCFS1 using proteomic analysis of secreted proteins. To investigate the consequences of the *lgt* mutation in host-cell interaction, the capacity of mutant and wild-type bacteria to stimulate TLR2 signaling and inflammatory responses was compared using (reporter-) cell-based models. These experiments exemplified the critical contribution of the acyl chains of lipoproteins in immunomodulation. To the best of our knowledge, this is the first study that investigated collective lipoprotein functions in a model strain for probiotic lactobacilli, and we show that the lipoproteins in *L. plantarum* WCFS1 are critical drivers of anti-inflammatory host responses toward this strain.

## Introduction

**FIGURE 1:**
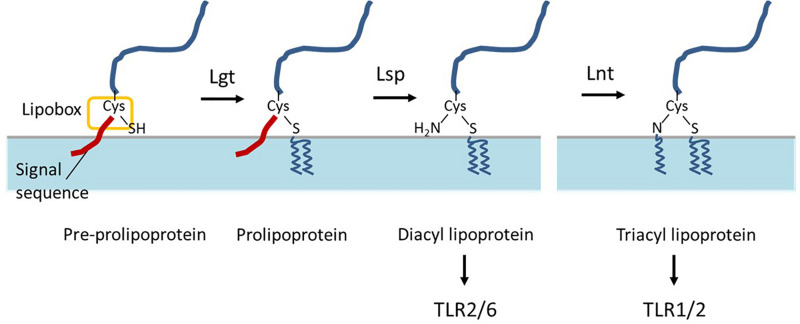
Schematic representation of bacterial lipoprotein biogenesis. After export across the cell membrane, pre-prolipoproteins undergo lipid modification by prolipoprotein diacylglyceryl transferase (Lgt) which transfers a diacylglyceryl moiety onto the cysteine residue in the lipobox, and results in prolipoproteins. Subsequently, lipoprotein signal peptidase (Lsp) cleaves of signal peptide at the direct N-terminal of the lipid-modified cysteine residue and results in diacyl lipoproteins. In some bacteria, lipoprotein N-acyl transferase (Lnt) adds a third acyl chain to the free amino group of the lipidated cysteine. The di- and tri-acyl lipoproteins produced by bacteria are differentially recognized by distinct TLR-2 heterodimers TLR2/6 and TLR1/2, respectively.

Bacterial lipoproteins are proteins that are post-translationally modified by acyl-conjugation, which anchors the protein on the extracellular face of the cytoplasmic membrane. These lipoproteins contain a typical N-terminal signal sequence that ends with the conserved [L/V/I]-[A/S/T]-[G/A]-C motif that is designated “lipobox” (Schenk et al., 2009). After export across the cell membrane, these lipoprotein precursors undergo their lipid modification, which is catalyzed by three conserved enzymes, following a mechanism that was first established in *Escherichia coli* (Braun and Wu, 1994). As a first step, the prolipoprotein diacylglyceryl transferase (Lgt) transfers a diacylglyceryl moiety onto the indispensable cysteine residue in the lipobox (Braun and Wu, 1994), which is targeted by the lipoprotein signal peptidase (Lsp) that cleaves of the signal sequence directly N-terminally of the lipid-modified cysteine residue. In the third step, lipoprotein *N*-acyl transferase (Lnt) adds a third acyl chain to the free amino group of the lipidated cysteine (Figure 1). In Gram-negative bacteria, the third step is essential for the release and transport of lipoproteins from the cytoplasmic membrane to the outer membrane, but the *E. coli*-type Lnt enzyme appears to be absent in low-GC-content Gram-positive bacteria of the Firmicutes phylum (Robichon et al., 2005; Hutchings et al., 2009). Nevertheless, tri-acylated lipoproteins have been reported for bacteria belonging to this phylum, including *Staphylococcus aureus* and *Streptococcus pneumoniae*, suggesting the presence of an unrecognized Gram-positive *N*-acyltransferase function (Kurokawa et al., 2009; Asanuma et al., 2011; Bartual et al., 2018).

Lipoproteins are involved in diverse biological functions. Many lipoproteins function as substrate binding proteins (SBPs) of ATP-binding cassette (ABC) transporters involved in import of a variety of substrates, such as sugars, metal ions, amino acids, oligopeptides, and nucleotides (Hutchings et al., 2009). SBPs provide high affinity substrate binding and delivery to the membrane permease components (Biemans-Oldehinkel et al., 2006), which is important for nutrient uptake and may also play a role in environmental sensing (Dartois et al., 1997; Hoskisson and Hutchings, 2006; Hutchings et al., 2006). Besides their roles in import, lipoproteins in various Gram-positive bacteria are also involved in modulation of two-component signal transduction systems, cell envelope stability, adhesion, protein secretion and folding, or electron transfer processes at the cell membrane that support oxidative and antibiotic stress tolerance (Abdullah et al., 2014; Ezraty et al., 2017; Hutchings et al., 2009; Kohler et al., 2016; Nguyen and Gotz, 2016; Saleh et al., 2013; Shahmirzadi et al., 2016). Several studies also reported the role of lipoproteins in virulence, where these molecules contribute to colonization, invasion, immune evasion and survival capacities of pathogens in the host (Nguyen and Gotz, 2016). Many studies of the function of lipoproteins in pathogenic bacteria involved the analysis of mutants deficient in essential functions in lipoprotein biogenesis like *lgt* or *lsp*, typically leading to reduced adhesion and internalization and/or to attenuation of virulence in animal infection models (Kovacs-Simon et al., 2011; Nguyen and Gotz, 2016).

By contrast, the role that lipoproteins play in the physiology and host interaction of probiotic bacteria has not been reported in much detail. Probiotics are defined as live microorganisms that, when administered in adequate amounts, confer a health benefit on the host (FAO and WHO, 2002). The molecular interaction between probiotics and the host are proposed to play a key role in the health-promoting effects associated with these bacteria (Lebeer et al., 2008; Bron et al., 2011; [Bibr B38][Bibr B29]). Bacterial lipoproteins are well-recognized microorganism-associated molecular patterns (MAMPs), which interact with Toll-like receptor (TLR) 2, an important pattern recognition receptor (PRR) of the host innate immune system (Brightbill et al., 1999; Underhill and Ozinsky, 2002). Moreover, the di- and tri-acyl lipoproteins produced by bacteria are differentially recognized by distinct TLR-2 heterodimers TLR2/6 and TLR1/2, respectively ([Bibr B55][Bibr B23]; Kang et al., 2009). Although it was initially thought that TLR2 heterodimers with TLR1 or TLR6 merely expanded the repertoire of bacterial ligand recognition (Farhat et al., 2008), it has since then been demonstrated that the magnitude of activation as well as the downstream signal transduction cascades are distinct for TLR1/2 and TLR2/6 heterodimers (Couture et al., 2012; Liu et al., 2012; Rolf et al., 2015), indicating their distinct role in innate signaling.

Deletion of *lgt* provides a means to study the general role of all lipoproteins in bacterial physiology and immunomodulation. Here we describe the impact of *lgt* mutation in the probiotic model strain *Lactobacillus plantarum* WCFS1 (Kleerebezem et al., 2003). We demonstrate that Lgt impacts on the secreted proteome, and specifically leads to the release of predicted lipoproteins that remain non-acylated. Furthermore, the capacity of the *L. plantarum lgt* mutant to stimulate TLR2 signaling and inflammatory responses was compared to those of the wild-type, illustrating the contribution of the lipoprotein acyl chains to immunomodulation. Although it has previously been established that lipoproteins are among the main TLR2 signaling ligands, to the best of our knowledge, this is the first study that explored the role of lipoproteins in immunomodulation by a model species of the probiotic lactobacilli.

## Materials and Methods

### Bacterial Strains and Culture Conditions

Bacterial strains used in this work are listed in Table 1. *Lactobacillus plantarum* WCFS1 and its derivatives were grown at 37°C in MRS broth (Difco, West Molesey, United Kingdom) or in 2-fold concentrated chemically defined medium [2xCDM, (Teusink et al., 2005)], without aeration. *Escherichia coli* strain TOP10 (Invitrogen, Bleiswijk, Netherlands) was used as an intermediate cloning host, and was grown at 37°C in TY broth (Killmann et al., 2002) with aeration (Sambrook et al., 1989). Solid media were prepared by addition of 1.5% (w/v) agar to the broths. Antibiotics were added where appropriate and concentrations used for *L. plantarum* and *E. coli* strains were 10 μg/ml chloramphenicol (Cm), and 30 and 200 μg/ml erythromycin (Ery), respectively.

**TABLE 1:** Bacterial strains, plasmids, and primers used in this study.

Strains	Characteristics^a^	References
***L. plantarum***		
WCFS1	Single-colony isolate of *L. plantarum* NCIMB8826. Isolate from human saliva, United Kingdom.	Kleerebezem et al., 2003
NZ3400Cm	Cm^r^; WCFS1 derivative; chromosomal integration of *cat* cassette into H-locus	Remus et al., 2012
NZ3565Cm (Δ*lgt*)	Cm^r^; derivative of WCFS1 containing a *lox66*-P_32_-*cat*-*lox71*-tag6.6 replacement of *lgt (lp_0755)* (*lgt*::*lox66*-P_32_-*cat*-*lox71*- tag6.6)	This work
***E. coli***		
TOP 10 *end*A1 *nup*G	Cloning host; F^–^ *mcr*A Δ(*mrr*-*hsd*RMS-*mcr*BC) φ80*lac*ZΔM15 Δ*lac*X74 *rec*A1 *ara*D139 Δ(*ara*-*leu*)7697 *gal*U *gal*K *rps*L (Str^R^)	Invitrogen

**Plasmids**	**Descriptions^a^**	**References**

pNZ5319	Cm^r^ Ery^r^; Mutagenesis vector for gene replacements in *L. plantarum*	Lambert et al., 2007
pNZ3565	Cm^r^ Ery^r^; pNZ5319 derivative containing homologous regions up- and downstream of *lgt* (*lp_0755*)	This work

**Primers**	**Sequence^b^**	**References**

is128 tag-lox66-F3	5′-AAATCTACCGTTCGTATAATGTATG-3′	Bron et al., 2012
is129 tag-lox71-R3	5′-CTCATGCCCGGGCTGTAACCG-3′	Bron et al., 2012
IS169	5′-TTATCATATCCCGAGGACCG-3′	van Bokhorst-van de Veen et al., 2012
87	5′-GCCGACTGTACTTTCGGATCC-3′	van Bokhorst-van de Veen et al., 2012
CreF	5′-CGATACCGTTTACGAAATTGG-3′	van Bokhorst-van de Veen et al., 2012
CreR	5′-CTTGCTCATAAGTAACGGTAC-3′	van Bokhorst-van de Veen et al., 2012
EryintF	5′-TCAAATACAGCTTTTAGAACTGG-3′	van Bokhorst-van de Veen et al., 2012
EryintR	5′-ATCACAAACAGAATGATGTACC-3′	van Bokhorst-van de Veen et al., 2012
lgt-up-F	5′-TTTGGCAGGAAGTGTAACCG-3′	This work
lgt-up-R	5′-GCATACATTATACGAACGGTAGATTTATTCACGCTACTGCCATCTCC-3′	This work
lgt-down-F	5′-CGGTTACAGCCCGGGCATGAGGCAGAAAATAAGTAGATTAGAGG-3′	This work
lgt-down-R	5′-AATCTCAGGTTTCCCCTCGC-3′	This work
lgt-out-F	5′-AAGTGTGGCCGCTTGAAAGGG-3′	This work
lgt-out-R	5′-AACATTTCTTTAGGCATCGCC-3′	This work

*^*a*^Cm^*r*^, chloramphenicol resistant; Ery^*r*^, erythromycin resistant. ^*b*^Underlined nucleotides indicate parts of the primers that are complementary to the is128-lox66-F3 and is129-lox71-R3 primers.*

### DNA Manipulations

Plasmids and primers used are listed in Table 1. Standard procedures were used for DNA manipulations in *E. coli* (Sambrook et al., 1989). Plasmid DNA was isolated from *E. coli* using a JETSTAR kit (Genomed GmbH, Bad Oberhausen, Germany). *L. plantarum* DNA was isolated as described previously (Josson et al., 1989). PCR amplifications were performed using hot-start KOD DNA polymerase (Novagen, Madison, WI, United States). Amplicons were purified using WizardSV Gel and PCR Clean-Up System (Promega, Leiden, Netherlands). Restriction endonucleases (Fermentas GmbH, St. Leon-Rot, Germany), MSB Spin PCRapace (Invitek GmbH, Berlin, Germany), PCR Master Mix (Promega) and T4 DNA ligase (Invitrogen) were used as specified by the manufacturers.

### Construction of *lgt* Deletion Strain

The *lgt* deletion mutant was constructed as described previously (Lambert et al., 2007), using a double crossing-over strategy to replace the *lgt* gene by a chloramphenicol resistance cassette (*lox66*-P_32_*cat*-*lox71*) (Lambert et al., 2007). In this study, a derivative of the mutagenesis vector pNZ5319 (Lambert et al., 2007), designated pNZ5319TAG was used to introduce a unique 42-nucleotide tag into chromosome during gene deletion, which can be used for mutant tracking purposes in mixed populations (not relevant for the study presented here). The upstream and downstream flanking regions of *lgt* (*lp_0755*) gene were amplified by PCR using the primer pairs lgt-up-F/R and lgt-down-F/R primers, respectively (Table 1). The amplicons generated were joined by a second PCR to *lox*66-P_32_*cat*-*lox*71-tag by a splicing by overlap extension strategy (Horton, 1993), using lgt-up-F/lgt-down-R primers. The resulting PCR products were digested with *Swa*I and *Ecl*136II, and cloned into similarly digested pNZ5319TAG. The obtained mutagenesis plasmids were transformed into *L. plantarum* WCFS1 as described previously (Josson et al., 1989). The resulting transformants were assessed for a double cross over integration event by selecting for Cm resistance and Ery sensitivity. The selected colonies were further confirmed by PCR using targets-out-F/R primers (Table 1). A single colony displaying the anticipated antibiotic resistance phenotype and genotype was selected, yielding NZ3565Cm (*L. plantarum* WCFS1 Δ*lgt*).

### Isolation of Released Proteins and SDS-PAGE

For the isolation of proteins released into the culture supernatants, *L. plantarum* WCFS1 and its Δ*lgt* derivative were grown overnight to an OD_600_ of approximately 5 in 100 mL of 2xCDM. The culture supernatants were filtered through a hydrophilic polyvinylidene fluoride (PVDF) filter (0.22 μm pore size, 25 diameter; Millex Millipore, United States) to remove any remaining bacterial cells, and proteins were precipitated by adding trichloroacetic acid (TCA) to a final concentration of 16%, followed by an overnight incubation at 4°C. The precipitated proteins were pelleted by centrifugation at 16000 × *g* for 15 min. The protein pellets were washed with 200 μl acetone and then air-dried at 50°C. Dried protein pellets were solubilized in NuPAGE loading buffer and dithiothreitol (DTT) reducing agent (both from Invitrogen). The samples of released proteins were visualized by SDS-PAGE using the NuPAGE electrophoresis system with NuPAGENovex 4–12% Bis-Tris gels with MOPS SDS running buffer (Invitrogen), followed by Coomassie brilliant blue staining using standard procedures (Sambrook et al., 1989) and overnight destaining in Milli-Q water.

### Sample Preparation for Mass Spectrometry

For in-gel trypsin digestion, the protein-containing SDS-PAGE gel was reduced with 10 mM dithiotreitol (DTT) in 50 mM ammonium bicarbonate (ABC) for 1 h at 60°C, followed by alkylation with 20 mM iodoacetamide in 100 mM Tris buffer (pH 8.0) in the dark for 1 h at room temperature. After thorough washing in Milli-Q water, the gel lane of each sample was divided into five slices that were individually cut into small pieces (ca. 1 mm^3^). The gel pieces were transferred to protein LoBind tubes (Eppendorf, Hamburg, Germany) for all following procedures to minimize protein loss. Sample were freeze-thawed to increase enzyme accessibility of the gel pieces, and incubated in ABC buffer containing 5 ng/μL Bovine Sequencing Grade Trypsin (Roche) for 2 h at 45°C. The solution was sonicated briefly (1 s) and was adjusted to an pH of approximately 2.0 with 10% trifluoroacetic acid (TFA).

The trypsin-digested samples were further cleaned up to remove any gel residues using C18 microcolumns as described previously (Zhang et al., 2015). In short, C18 microcolumns were prepared in 200-μL Eppendorf tips by placing a small piece (ca. 1 mm in diameter) of a C18 Empore disk and then applying 4 μL of 50% slurry of LiChroprep C18 column material in methanol. The microcolumns were washed twice with 200 μl methanol and subsequently equilibrated with 100 μl of 1 ml/l formic acid (HCOOH). The samples were applied to the microcolumns and washed with 1 ml/l HCOOH. Samples were eluted using 50 μl of 50% acetonitrile/30% 1 ml/l HCOOH into clean LoBind tubes. The sample volume was then reduced in a vacuum concentrator (Eppendorf Vacufuge) at 45°C for 20 to 30 min until a volume below 20 μl was reached.

The liquid-chromatography tandem mass spectrometry (LC-MS/MS) analysis was performed on a Proxeon EASY-nLC system (Thermo Scientific) coupled with a LTQ Orbitrap XL mass spectrometer (Thermo Scientific). The chromatographic separation was performed on a combination of a Prontosil 300-5-C18H pre-concentration column with a Prontosil 300-3-C18H analytical column (Bischoff Chromatography, Leonberg, Germany) (Kessels et al., 2014). For peptide identification, the protein reference database of *Lactobacillus plantarum* (strain ATCC BAA-793/NCIMB 8826/WCFS1) for peptides and proteins identification downloaded from UniProt^1^ was used. A set of 31 protein sequences of common contaminants was added including Trypsin (P00760, bovine), Trypsin (P00761, porcine), Keratin K22E (P35908, human), Keratin K1C9 (P35527, human), Keratin K2C1 (P04264, human), and Keratin K1C1 (P35527, human). Label-free quantitation (LFQ) of detected proteins was calculated by MaxQuant algorithm to compare quantity cross samples. Relative abundances were calculated by the ratio of LFQ of detected peptides in wild-type and *lgt* mutant and presented in log_10_ value.

### Toll-Like Receptor (TLR) Assay

Human embryonic kidney (HEK)-293 TLR reporter cell lines expressing human TLR1/2, TLR2/6, or TLR4, harboring pNIFTY, a NF-κB luciferase reporter construct (Invivogen, Toulouse, France) (Karczewski et al., 2010), were used. The HEK-293 reporter cell lines were seeded at 6 × 10^4^ cells/well in 96-well plates and incubated overnight under standard culture conditions. Cells were then stimulated with late-stationary bacterial cultures of the *L. plantarum* NZ3400Cm, a *L. plantarum* WCFS1 derivative with a chromosomal integration of the *cat* cassette in a neutral chromosomal locus (Remus et al., 2012), and *lgt* deletion strain (NZ3565Cm) at a multiplicity of infection (MOI) of 1:10, HEK cell to bacteria. The TLR1/2 agonist Pam3CSK4 (5 μg/mL, Invivogen) and TLR2/6 agonist Pam2CSK4 (5 μg/mL, Invivogen) were used as positive controls and PBS served as the negative control.

### Peripheral Blood Mononuclear Cells (PBMC) Assay

The assay was performed as described previously (van Hemert et al., 2010) and was approved by Wageningen University Ethical Committee and was performed according to the principles of the Declaration of Helsinki. Peripheral blood of healthy donors was from the Sanquin Blood Bank, Nijmegen, Netherlands. PBMCs were separated from the blood using Ficoll-Paque Plus gradient centrifugation according to the manufacturer’s description (Amersham biosciences, Uppsala, Sweden). The mononuclear cells were collected, washed in Iscove’s Modified Dulbecco’s Medium (IMDM) + glutamax (Invitrogen, Breda, Netherlands) and adjusted to 1 × 10^6^ cells/ml in IMDM + glutamax supplemented with penicillin (100 U/ml) (Invitrogen), streptomycin (100 μg/ml) (Invitrogen), and 1% human AB serum (Lonza, Basel, Switzerland). PBMCs (1 × 10^6^ cells/well) were seeded a night prior to the experiment in 48-well tissue culture plates and incubate at 37°C in 5% CO_2_. Bacteria from late-stationary phase were added to PBMCs at a MOI of 1:10 (PBMC to bacteria) PBMCs from 3 different donors were used in the assay. Following 24 h incubation at 37°C in 5% CO_2_, culture supernatants were collected and stored at −20°C prior to cytokine analysis. Cytokines were measured using a FACSCanto II flow cytometer (BD Biosciences, New Jersey, United States) and BD Cytometric Bead Array Flexsets (BD Biosciences) for interleukin (IL)10 and IL12p70, Tumor Necrosis Factor (TNF)α, IL6, IL1β, and IL8 according to the manufacturer’s procedures. Concentrations of cytokines were calculated based on the standard curves in the BD Biosciences FCAP software.

### Statistical Analysis

The TLR and PBMC assays were performed in triplicate. One-way ANOVA followed by Tukey’s multiple comparison correction was used to compare TLR2 activations between strains. The paired *t*-test was used to determine the Log values of PBMCs cytokine production after stimulated with wild-type verse mutant strains for individual donors. GraphPad Prism 5 software (GraphPad Software, San Diego, CA, United States) was used for all determinations, and a *P* value < 0.05 was considered significant. Notably, the parametric statistical analyses applied are justified on basis of the normal data distribution of measurements obtained in these type of analyses, which is based on evaluation of data distribution on numerous datasets obtained in similar assays in our laboratory (data not shown).

### Data Availability

The mass spectrometry proteomics data have been deposited to the ProteomeXchange Consortium via the PRIDE (Vizcaino et al., 2016) partner repository with the dataset identifier PXD019218.

## Results

### Construction of Lgt-Deficient *L. plantarum* WCFS1

Prolipoprotein diacylglyceryl transferase (Lgt) is the key enzyme for lipidation in lipoprotein biosynthesis, where it catalyzes the transfer of a diacylglyceryl moiety onto a conserved cysteine in the lipobox of prolipoproteins (Hutchings et al., 2009). The gene annotated to encode this function (*lgt*; *lp_0755*) in the *L. plantarum* WCFS1 genome (Kleerebezem et al., 2003) was mutated by double cross-over gene replacement of the *lgt* coding region by a chloramphenicol acetyltransferase (*cat*) cassette (Lambert et al., 2007), resulting in an *lgt* deficient derivative of strain WCFS1, designated NZ3565Cm (Δ*lgt*). This *lgt*-mutant strain enables the study of the generic impact of lipoproteins on physiological and immunomodulatory properties of this model probiotic-bacterium.

Under laboratory conditions, the growth and cell-morphology of the *lgt* deletion mutant were undistinguishable from those of the wild-type strain (data not shown). This is in agreement with earlier observations that suggested that although Lgt is essential in Gram-negative bacteria, it appears to be dispensable in Gram-positive bacteria grown under laboratory conditions (Hutchings et al., 2009).

### Lgt Is Important for Membrane Anchoring of Lipoproteins

**FIGURE 2:**
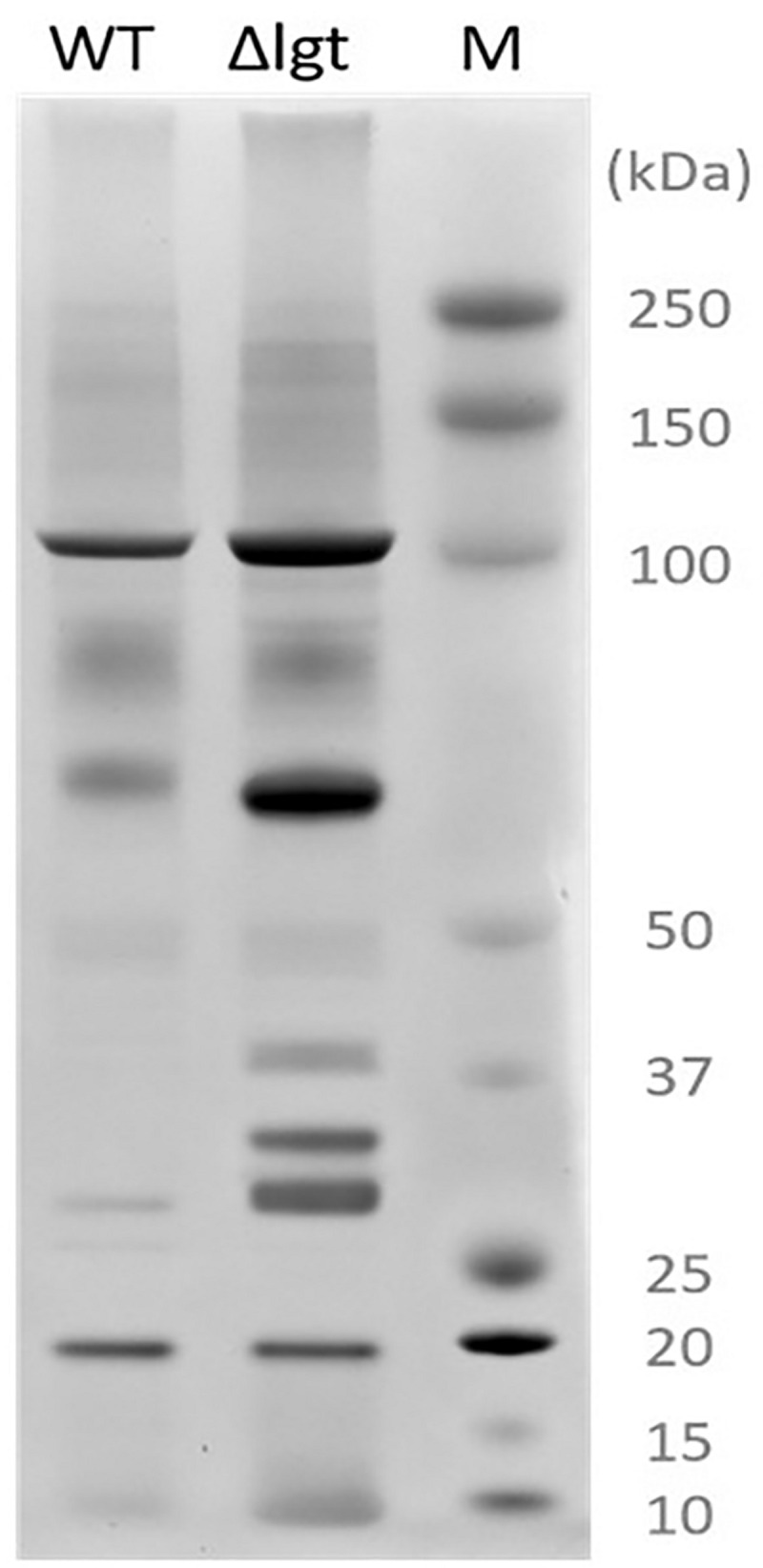
Secreted proteins extracted from *L. plantarum* WCFS1 (WT) and its *lgt* deletion derivative (Δ*lgt*; NZ3565Cm). Proteins were separated by SDS-PAGE and visualized by Coomassie blue staining. On the right side, the protein size marker (M), Precision Plus Protein Dual Color Standards (Bio-Rad).

**FIGURE 3:**
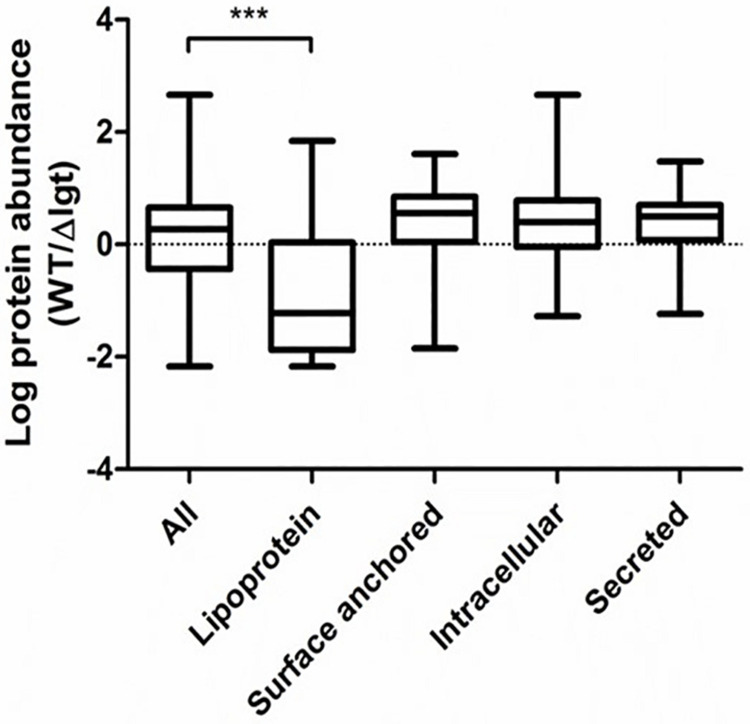
Relative abundance of secreted proteins in *L. plantarum* WCFS1 wild*type (WT) and the *lgt* deletion strain (Δ*lgt*; NZ3565Cm). The relative abundance is defined as the ratios in the label free quantitation (LFQ, log_10_ value) of detected proteins in wild-type and the deletion strain in tandem mass spectrometry (MS/MS) analysis. The abundance was compared with all proteins detected in both samples, or within specific protein groups. The ratios from specific proteins groups were compared against all proteins to test for significant differences using one-way ANOVA followed by Tukey’s multiple comparison correction and significant differences are indicated; ***P ≤ 0.001.

We investigated the impact of *lgt* deletion on the membrane-anchoring of lipoproteins, by comparing the supernatants of wild-type and the mutant using SDS-PAGE. The protein-pattern observed revealed a clear difference between the supernatant of the wild-type strain and *lgt* mutant, which released a much higher abundance of proteins into its culture medium as compared to its parental strain (Figure 2). The SDS-PAGE gel containing supernatant proteins were subjected to in-gel trypsin digestion, followed by tandem mass spectrometry (MS/MS) analysis to identify the released proteins. There are 7 and 9 proteins that are exclusively detected in the supernatant of wild-type and *lgt* mutant cultures, respectively (Supplementary Table S1). The protein abundances of these proteins were set to detection limit to enable the calculation of the relative protein abundance of the proteins detected in both the wild-type and *lgt* mutant culture supernatants. The overall protein-abundance in the culture supernatant of the wild-type and its *lgt* derivative appeared to be similar, but the relative abundance of predicted lipoproteins was significantly higher in the supernatant of the *lgt* deficient strain compared to other classes of secreted proteins (Figure 3, Supplementary Figure S1, and Supplementary Table S1). Many of the lipoproteins found in higher abundance in the supernatant of *lgt* mutant belong to the predicted substrate binding proteins of ATP-binding cassette (ABC) transporters associated with various substrates, including iron, phosphate, amino acids, maltose, and maltodextrin (Supplementary Table S1). The proteome analysis detected 38 out of the 47 predicted lipoproteins encoded by the WCFS1 genome (Kleerebezem et al., 2010), thereby broadly representing this group of proteins. These results confirm the importance of Lgt in appropriate anchoring of lipoproteins in the cytoplasmic membrane.

### Acyl Chains of Lipoproteins Are Important for TLR1/2 Signaling Capacity of *L. plantarum* WCFS1

**FIGURE 4:**
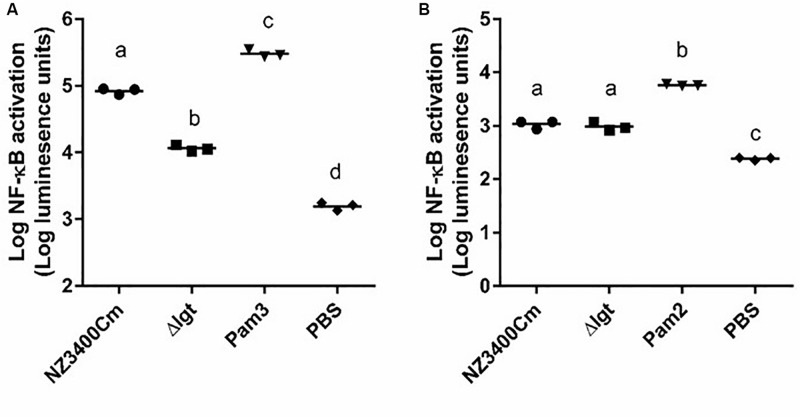
TLR1_2 and TLR2_6 signaling capacities of NZ3400Cm, a *L. plantarum* WCFS1 derivative with a chromosomal integration of the *cat* cassette in a neutral chromosomal locus (NZ3400Cm, Table 1) and the *lgt* deletion mutant, NZ3565Cm (Δ*lgt*; NZ3565Cm). TLR1_2 **(A)** and TLR2_6 **(B)** activation were determined using TLR-expressing HEK cell lines, containing a NF-kB responsive luciferase reporter system. Measurements were performed in triplicate and are presented as log_10_ luminescence units, and individually displayed (*n* = 3), while the bar indicates the median. PBS serves as a negative control, while Pam3CysSK4 (Pam3) and Pam2CysSK4 (Pam2) are the positive stimulus of TLR1_2 **(A)** and TLR2_6 **(B)** activation, respectively. Data comparison of the wild-type and the deletion derivative was tested for significant differences using one-way ANOVA followed by Tukey’s multiple comparison correction and samples with significant different NF-kB activation are indicated with different letters.

The human innate immune system has been reported to recognize bacterial lipoproteins by TLR1/2 and TLR2/6 heterodimers that recognize tri- and di-acylated lipoproteins, respectively (Schenk et al., 2009). The impact of *lgt* deletion in *L. plantarum* on TLR2 heterodimer signaling was investigated using established HEK-293 reporter cell lines that express human TLR1/2 or TLR2/6 heterodimers combined with a NF-κB promoter-controlled luciferase gene. The synthetic agonists Pam3CSK4 and Pam2CSK4 were used as positive controls for TLR1/2 and TLR2/6 activation, respectively. The wild-type strain *L. plantarum* NZ3400Cm (Remus et al., 2012) stimulated both TLR1/2 and TLR2/6 signaling at a moderate level (Figures 4A,B, respectively). The *lgt* deletion strain, Δ*lgt* (NZ3565Cm), stimulated significantly lower TLR1/2 signaling as compared to the wild-type strain (Figure 4A), whereas its capacity to stimulate TLR2/6 signaling appeared to be unaffected as compared to the wild-type strain (Figure 4B). The observation that the *lgt* mutant of *L. plantarum* WCFS1 affected TLR1/2 signaling and not TLR2/6 signaling, suggests that tri-acylated lipoproteins are dominant in this strain.

### Lipid Moiety of Lipoproteins Is Important for Anti-inflammatory Properties of *L. plantarum* WCFS1

**FIGURE 5:**
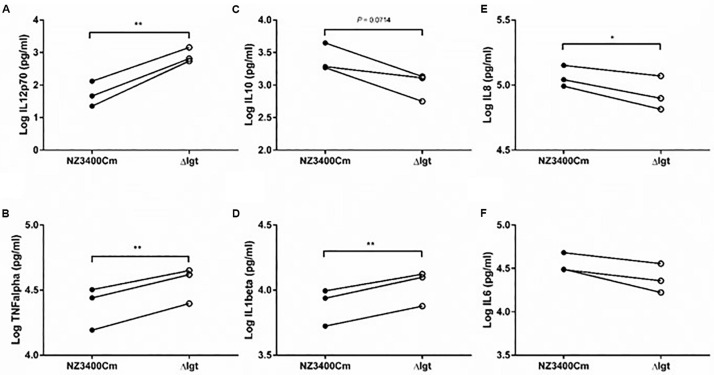
Immunomodulatory effect of NZ3400Cm, a *L. plantarum* WCFS1 derivative with a chromosomal integration of the *cat* cassette in a neutral chromosomal locus (NZ3400Cm, Table 1), and the *lgt* deletion strain NZ3565Cm (Δ*lgt*; NZ3565Cm). Cytokine production was determined in human PBMCs (*n* = 3 donors) after 24 h co-incubation with the bacterial cells. The IL12 **(A)**, TNFα **(B)**, IL10 **(C)**, IL1β **(D)**, IL8 **(E)**, and IL6 **(F)** cytokine production levels are presented as Log values. The cytokine levels for individual donors stimulated with the strains were connected by a line to focus the read-outs on changes elicited by the deletion. Significant differences between cytokine levels induced by wild-type strains and their corresponding mutants (paired *t*-test) are indicated; *P ≤ 0.05; **P ≤ 0.01; the *P* value of the difference of IL10 production by the NZ3400Cm and Δ*lgt* strains is indicated in the corresponding panel **(C)**.

Although many studies have shown that Gram-positive pathogens that lack Lgt activity display attenuated immune activation or virulence, little is known about the effect of this phenotype in probiotic bacteria. We explored the impact of *lgt* deletion on general immune responses using cytokine production by human peripheral blood mononuclear cells (PBMCs). The *lgt* deletion mutant stimulated a more pro-inflammatory responses in PBMCs as compared to the wild-type strain (NZ3400Cm), including a higher production of the pro-inflammatory cytokines, IL12, TNFα, IL1β, and IL8 (Figures 5A,B,D,E, respectively). Moreover, the *lgt* mutant strain tends to induce lower levels of production of the anti-inflammatory cytokine IL10 relative to the wild-type (Figure 5C). As a consequence, the IL10/IL12 ratio, which has been reported as an indicator for *in vivo* performance in a mouse colitis model (Foligne et al., 2007), is significantly lower in *lgt* mutant than the wild-type (Supplementary Figure S2), implying a more pro-inflammatory profile after exposure to the mutant. These results illustrate the importance of Lgt and lipoproteins in the overall immunomodulatory properties associated with *L. plantarum* WCFS1, and in particular exemplify the contribution of lipoproteins in the anti-inflammatory properties in *L. plantarum* WCFS1.

## Discussion

Diacylglyceryl transferase is an essential enzyme in various Gram-negative bacteria (Hutchings et al., 2009). Since many lipoproteins in Gram-negative bacteria are localized at the outer membrane, defects in lipoprotein biosynthesis will cause mislocalization and/or accumulation of the precursors in the periplasmic space, which has been reported to be lethal to the cells (Matsuyama et al., 1995; Robichon et al., 2005). In contrast, Lgt-enzymes appear dispensable in a variety of Gram-positive bacteria (Hutchings et al., 2009). In agreement with this notion, the *lgt* deletion derivative of *L. plantarum* displayed normal cellular morphology and growth characteristics. The limited impact of *lgt* deletion in Gram-positive bacteria may be explained by the different impact of the loss of Lgt function on the subcellular location of lipoproteins. In Gram-negative bacteria many lipoproteins are targeted to the outer membrane, and the defective biogenesis of these proteins due to Lgt deficiency may lead to protein accumulation and clogging of the periplasm. In contrast, in Gram-positive bacteria non-acylated lipoproteins tend to remain functionally localized in the cell wall, or are released into the medium. The functional localization of non-acylated lipoproteins in Gram-positive bacteria is supported by the observation that an *lgt* mutation is not lethal in *Bacillus subtilis*, although at least the lipoprotein PrsA fulfills an essential role in this bacterial species (Leskela et al., 1999). In *L. plantarum* deletion of *lgt* led to mislocalization and release of a range of lipoproteins into the culture supernatant, including many substrate binding proteins (SBPs) associated with ABC transporters annotated to be involved in the import of amino acids, oligopeptides, maltose, and maltodextrin. Such mislocalization of these SBPs could reduce the efficiency of transport of the corresponding substrates, which may be reflected in changes in the corresponding metabolic processes. However, we did not observe differences in growth characteristics between the wild-type strain and its *lgt* derivative when cultured in minimal medium with maltose as a sole carbon source (Supplementary Figure S3). Besides this targeted phenotypic evaluation, further phenotype evaluations would be needed to pinpoint specific phenotype consequences of the *lgt* mutation and the corresponding mis-localization of the lipoprotein substrate binding proteins. Such experiments are likely to require the use of chemically defined minimal media to reliably limit the environmental availability of required nutrients, analogous to what was shown in *Staphylococcus aureus* (Stoll et al., 2005). This lack of consequence in growth characteristics of the *lgt* mutation, may be explained by a certain proportion of SBPs that remains associated with the corresponding transporters. Notably, it is unclear whether the lipoprotein signal peptidase (Lsp) is able to efficiently cleave all the non-lipid modified precursor lipoproteins in a *lgt* mutant strain, and failure to remove the signal peptide may retain lipoprotein precursors anchored in the cell membrane. Such retention mechanism is supported by studies that detected differently processed lipoprotein precursors can be detected in a Lgt-deficient background, including precursors containing their signal peptides or proteins cleaved by peptidases other than Lsp (Hutchings et al., 2009). Alternatively, it is also possible that cells can compensate for the lack of appropriately localized SBPs by elevated expression of the transport functions. Overall, our results establish that the *lgt* encoded function is dispensable for the laboratory-growth of *L. plantarum* WCFS1 and deletion of the *lgt* gene has a minimal impact on bacterial physiology under the conditions tested.

TLR2/6 and TLR1/2 heterodimers recognize di- or tri-acylated lipoproteins, respectively. Crystal structure analyses revealed that TLR1 has a hydrophobic pocket that enables the binding of the third acyl chain, which is lacking in TLR6 that can only accommodate di-acylated lipoproteins (Jin et al., 2007; Kang et al., 2009). The third acyl chain has been shown to be crucial for biosynthesis and biogenesis of lipoproteins in Gram-negative bacteria that are ending up in the outer membrane of these bacteria (Robichon et al., 2005), and appropriate subcellular localization (i.e., outer membrane biogenesis) of lipoproteins is essential in these bacteria. Moreover, since no orthologou genes of *lnt*, the gene responsible for transferring a third acyl chain on the N-terminal cysteine of lipoproteins, has been recognized in the genomes of low-GC-content Gram-positive bacteria, such as species belonging to the genera of *Bacillus*, *Lactobacillus*, *Listeria*, *Staphylococcus*, and *Streptococcus*, it has been long been assumed that these bacteria produce di-acyl lipoproteins (Nakayama et al., 2012). However, tri-acylated lipoproteins have been detected in several of these Gram-positive species, including *Staphylococcus aureus*, *Staphylococcus epidermidis*, and *Streptococcus pneumoniae* (Nguyen and Gotz, 2016), suggesting the presence of an *N*-acyltransferase in Gram-positive bacteria that displays insufficient similarity with the Gram-negative Lnt to be recognized as its functional equivalent (Asanuma et al., 2011; Kurokawa et al., 2009). Similarly, the reduction of specifically TLR1/2 signaling observed for the *L. plantarum lgt* mutant strain, implies that lipoproteins in this bacterial species are likely to be (predominantly) tri-acylated, or the N-acylation involves a long-chain fatty acid modification that are recognized through TLR1/2 rather than TLR2/6 (Nguyen et al., 2017). However, biochemical analysis would be required to verify the lipoprotein structures in *L. plantarum* WCFS1. The genome of this strain is annotated to encode three acyltransferases, *lp_0856*, *lp_0925*, and *lp_1181* that may be involved in an Lnt-like role in tri-acylation of lipoproteins. A fourth gene *lp_1916*, encoding a membrane protein, is not annotated as acyltransferase, but does contain a conserved acyltransferase domain of the acyltransferase family. Although one of these genes is potentially encoding the enzyme responsible for tri-acylation of lipoproteins in *L. plantarum* WCFS1, such function and its encoding gene remain to be established.

Since lipoproteins are established ligands of TLR2, an important PRR of the innate immune system, many studies have investigated the effect of *lgt* or *lsp* deletions on immune responses to, and virulence of Gram-positive pathogens (Hutchings et al., 2009; Nguyen and Gotz, 2016). Commonly, *lgt* or *lsp* deletions lead to attenuation of immune activation and/or reduced virulence of Gram-positive pathogens *in vitro* and *in vivo*, although some conflicting results have been reported (Nguyen and Gotz, 2016). For example, *lgt* and *lsp* deletion derivatives of *Streptococcus equi* (Hamilton et al., 2006) and *Streptococcus suis* (De Greeff et al., 2003), did not display attenuation in their natural hosts (pony and pig, respectively). Moreover, although a *Listeria monocytogenes lgt* mutant fails to activate TLR2 signaling it is significantly less virulent in a mouse infection model (Machata et al., 2008), whereas *lgt* mutants of *Streptococcus agalactiae* (Henneke et al., 2008) and *Staphylococcus aureus* (Bubeck Wardenburg et al., 2006; Mohammad et al., 2019) are hypervirulent in mouse infection models. These results imply a subtle and strain-specific balance between escaping protective immune defense related to loss of TLR2 activation and attenuated virulence by the loss of lipoprotein acylation in *lgt* mutants. Despite their important function as signaling molecules in microbe-host interactions, the role of lipoproteins in the immunomodulatory effect of probiotics has not been studied. Our results show that the *L. plantarum lgt* deletion derivative elicited more pro-inflammatory responses in PBMCs compared to its parental strain, suggesting that lipoproteins may act as important mediators of immune system recognition for probiotics where these molecules could drive more anti-inflammatory responses to such bacteria, which is relevant in the context of inducing tolerance. However, the exact mechanism (and diversity) behind lipoprotein mediated immune responses toward probiotics remains to be elucidated. Further studies focusing on purified lipoproteins could enable the controlled attenuation of pro-inflammatory immune responses in specific cell lineages (including monocytes and other fractions of the PBMC population), and may also be used to decipher their mechanistic interplay with pro-inflammatory pathways elicited by lipoprotein deficient strains. Moreover, structure-function studies of lipoproteins from pathogens and probiotics may unravel some key determinants involved in immune system recognition and activation, which may enable the host cells to distinguish harmful and beneficial bacteria (Nguyen et al., 2017).

Overall, the potentially important role of lipoproteins in probiotic function and in particular in their potential role in modulation of immune-responses is supported by the work presented here, and this role deserves further refined elucidation in terms of structure function correlation in the context of host-cell signaling by lipoproteins.

## Data Availability Statement

The raw data supporting the conclusions of this article will be made available by the authors, without undue reservation, to any qualified researcher.

## Author Contributions

I-CL, PB, and MK designed the study and wrote the manuscript. I-CL, IS, MM, NT, and MS executed the experimental work. SB and JV generated the proteome data. All authors agreed to the manuscript.

## Conflict of Interest

The authors declare that the research was conducted in the absence of any commercial or financial relationships that could be construed as a potential conflict of interest.

## Footnotes

1 http://www.uniprot.org/
