# Supplementary material for: Lipoproteins Contribute to the Anti-inflammatory Capacity of Lactobacillus plantarum WCFS1
Source: Front Microbiol. 2020 Jul 29;11:1822. doi: 10.3389/fmicb.2020.01822 (PMC7403179; doi:10.3389/fmicb.2020.01822)
Supplement: Supplementary file 1 [file Data_Sheet_1.pdf]

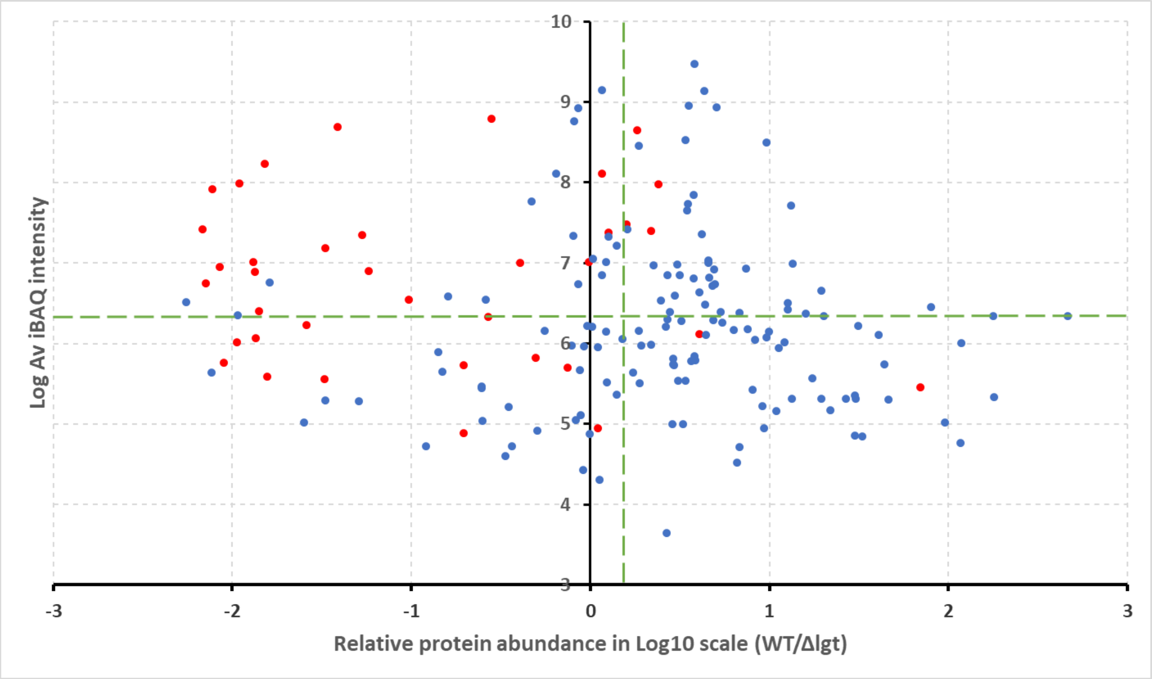

Figure S1. Relative abundance of secreted proteins in *L. plantarum* WCFS1 wild type and the *lgt* deletion strain (Δ*lgt*; NZ3565Cm). The relative abundance was the ratio taken from the label free quantitation (LFQ, log_{10} value) of detected proteins in wild type and the deletion strain in tandem mass spectrometry (MS/MS) analysis. The Av iBAQ intensity represents average Intensity-based absolute quantification of the protein detected in WT and *Δlgt*. The red dots represent lipoproteins, whereas blue dots represent all other type of proteins, average LFQ ratio and LogAv-iBAQ values are indicated by vertical and horizontal green dashed lines, respectively.

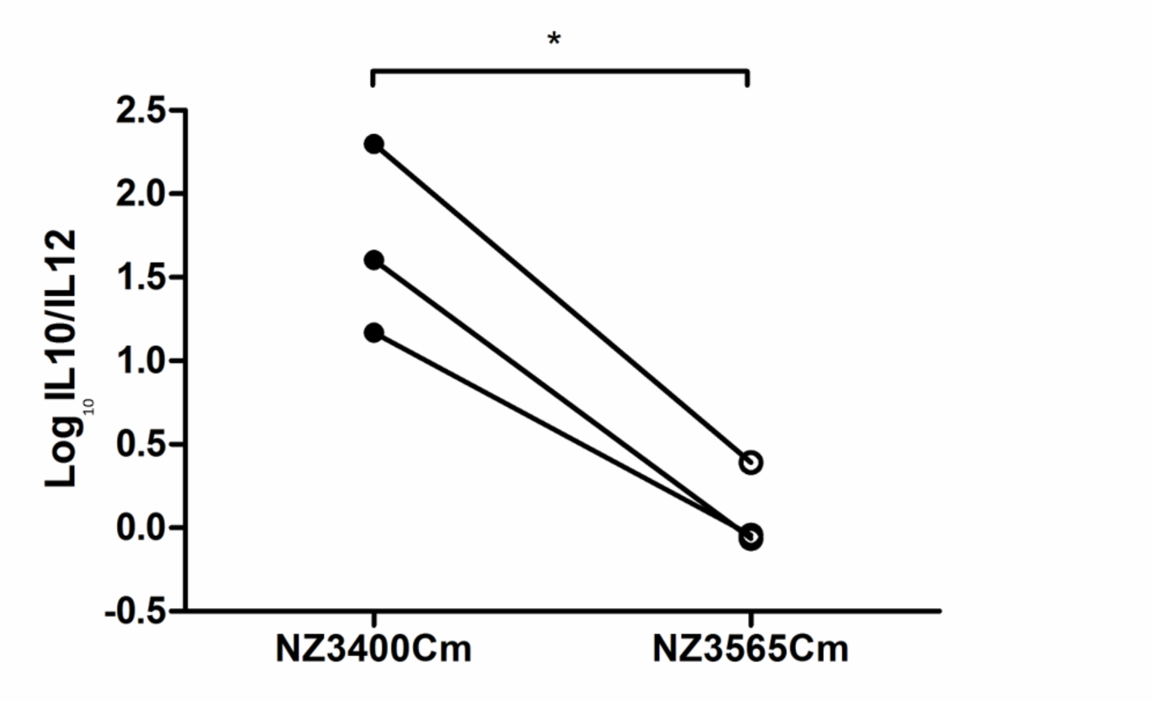

Figure S2. IL10/IL12 cytokine ratio (presented as log_{10} values) elicited by *L. plantarum* NZ3400Cm and the *lgt* deletion strain NZ3565Cm (Δ*lgt*; NZ3565Cm) following co-incubation with PBMC. The cytokine levels for individual donors stimulated with the strains were connected by a line to focus the read-outs on intrapersonal changes elicited by the deletion. The differences of IL10/IL12 ratio between NZ3400Cm and NZ3565Cm were statistically analyzed using paired t test; *, $P \leq 0.05$.

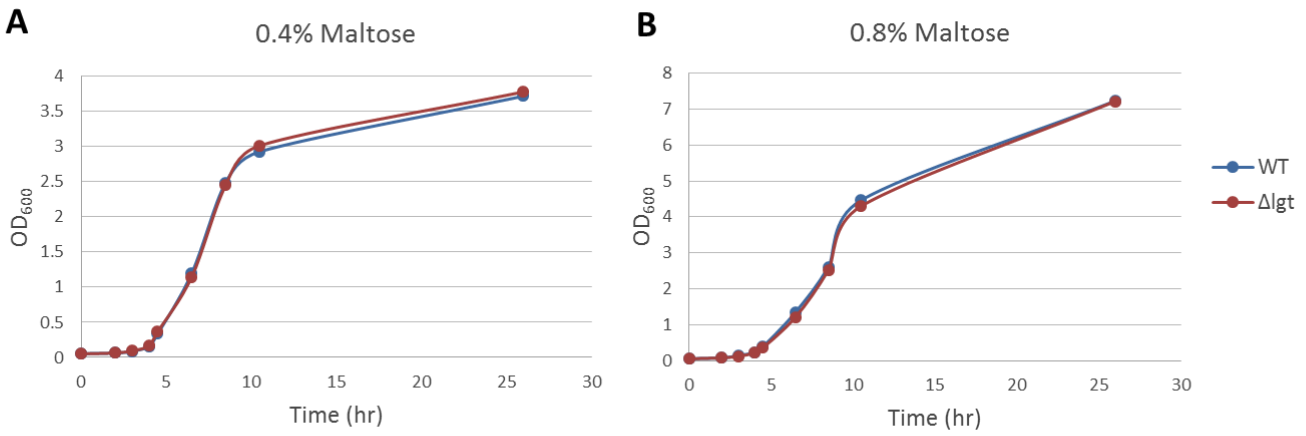

Figure S3. Comparison of the growth curves of *L. plantarum* NZ3400Cm and the *lgt* deletion strain (Δ*lgt*; NZ3565Cm). Bacteria were cultured in chemical defined medium with 0.4% (A) or 0.8% (B) maltose as a sole carbon source at 37° C. The growth was monitored for 26 hour by measuring the absorbance at 600 nm (OD_{600}).

Table S1. Proteins detected in mass spectrometry with different abundances between wild type *L. plantarum* WCFS1 and Δ*lgt* mutant

Gene ID	Relative abundance (WT/ Δlgt) [a]	Function	LocateP Subcellular Localization Prediction	Mol. weight [kDa]
**Only detected in Δ*lgt***				
lp_0200	-	ABC transporter, substrate binding protein	Lipid anchored	61.0
lp_0929	-	alkaline shock protein	Intracellular	15.8
lp_0937	-	membrane alanine aminopeptidase (aminopeptidase N)	Intracellular	93.9
lp_1539	-	lipoprotein precursor	Lipid anchored	21.1
lp_2974	-	ABC transporter, substrate binding protein	Lipid anchored	33.8
lp_3018	-	ABC transporter substrate binding protein	Lipid anchored	32.9
lp_3302	-	ABC transporter substrate binding protein	Lipid anchored	33.9
lp_3352	-	small heat shock protein	Intracellular	16.7
lp_3642	-	ABC transporter, substrate binding protein, mannose related oligosaccharides	Lipid anchored	54.1
**At least 2x higher in Δ*lgt***				
lp_0018	0.013	ABC transporter, substrate binding protein, oligopeptide	Lipid anchored	59.7
lp_0175	0.053	ABC transporter, substrate binding protein, maltodextrin	Lipid anchored	45.7
lp_0200	0.026	ABC transporter, substrate binding protein	Lipid anchored	61.0
lp_0201	0.008	ABC transporter, substrate binding proteins	Lipid anchored	61.1
lp_0733	0.033	phosphate ABC transporter, substrate binding protein	Lipid anchored	31.5
lp_0783	0.096	ABC transporter, substrate binding protein	Lipid anchored	61.1
lp_1010	0.281	serine-type D-Ala-D-Ala carboxypeptidase	Lipid anchored	33.0
lp_1070	0.013	lipoprotein precursor, FMN-binding protein	Lipid anchored	43.4
lp_1146	0.262	lipoprotein, pheromone precursor (putative)	Lipid anchored	108.0
lp_1452	0.015	peptidylprolyl isomerase	Lipid anchored	32.6
lp_1473	0.011	iron chelatin ABC transporter, substrate binding protein (putative)	Lipid anchored	21.1
lp_2312	0.195	ABC transporter, substrate binding protein, histidine	Lipid anchored	31.3
lp_2350	0.008	ABC transporter, substrate binding protein, D-Methionine -like precursor	Lipid anchored	29.9

lp_2397	0.497	prophage P2a protein 59; extracellular polysaccharide deacetylase, lipid-anchored (putative)	Lipid anchored	31.6
lp_2450	0.013	prophage P2a protein 7; extracellular protein with lipoprotein anchor	Lipid anchored	12.0
lp_2519	0.009	cell surface hydrolase, DUF915 family, membrane-bound (putative)	Lipid anchored	31.7
lp_2906	0.407	DNA-entry nuclease	Lipid anchored	34.3
lp_2974	0.016	ABC transporter, substrate binding protein	Lipid anchored	33.8
lp_2985	0.026	branched-chain amino acid ABC transporter, substrate binding protein	Lipid anchored	41.9
lp_3018	0.013	ABC transporter substrate binding protein	Lipid anchored	32.9
lp_3103	0.033	iron chelatin ABC transporter, substrate binding protein	Lipid anchored	34.1
lp_3209	0.011	ABC transporter, substrate binding protein, cystine	Lipid anchored	29.0
lp_3214	0.007	ABC transporter, substrate binding protein, cystathionine (putative)	Lipid anchored	29.3
lp_3302	0.068	ABC transporter substrate binding protein	Lipid anchored	33.9
lp_3642	0.053	ABC transporter, substrate binding protein, mannose related oligosaccharides	Lipid anchored	54.1
lp_0076	0.246	elongation factor G	Intracellular	77.0
lp_0764	0.336	Phosphoglucomutase	Intracellular	63.5
lp_0929	0.053	alkaline shock protein	Intracellular	93.9
lp_1046	0.472	ribosomal protein L24	Intracellular	11.4
lp_1879	0.246	DNA-binding protein	Intracellular	62.9
lp_3352	0.350	small heat shock protein	Intracellular	16.7
lp_1229	0.039	mannose-specific adhesion	LPxTG Cell-wall anchored	60.3
lp_0295	0.160	transport protein, MMPL family	Multi-transmembrane	143.3
lp_0925	0.149	Acyltransferase	Multi-transmembrane	15.8
lp_1000	0.014	transcriptional attenuator, cell envelope-related, LytR family	N-terminally anchored	28.2
lp_1097	0.269	manganese/zinc ABC transporter, substrate binding protein	Secretory(released)	42.6
lp_1278	0.058	serine-type D-Ala-D-Ala carboxypeptidase	Secretory(released)	38.6

**Only detected in WT**				
lp_0542	-	septum formation initiator	N-terminally anchored	15.2
lp_0575	-	mannose PTS, EIIAB	Intracellular	35.3
lp_1043	-	ribosomal protein L29	Intracellular	7.5
lp_1050	-	ribosomal protein S8	Intracellular	14.6
lp_1097	-	manganese/zinc ABC transporter, substrate binding protein	Secretory(released)	34.6
lp_3614	-	Oxidoreductase	Intracellular	33.4
lp_3686	-	ABC transporter, substrate binding protein	Lipid anchored	60.5
**At least 2x higher in WT**				
lp_2145	3.775	extracellular protein, DC-SIGN ligand	C-terminally anchored	45.8
lp_3678	3.872	cell surface protein precursor, DUF916 family	C-terminally anchored	39.6
lp_0355	3.309	multicopper oxidase (putative)	Intracellular	56.8
lp_0575	13.299	mannose PTS, EIIAB	Intracellular	35.3
lp_0578	2.867	non-ribosomal peptide synthetase NpsA	Intracellular	602.6
lp_0728	13.425	GroEL chaperonin	Intracellular	57.4
lp_0937	3.414	membrane alanine aminopeptidase (aminopeptidase N)	Intracellular	128.2
lp_1043	79.973	ribosomal protein L29	Intracellular	7.5
lp_1050	19.487	ribosomal protein S8	Intracellular	14.6
lp_1053	5.335	ribosomal protein S5	Intracellular	17.3
lp_1054	2.682	ribosomal protein L30	Intracellular	6.7
lp_1635	5.452	signal recognition particle protein Ffh	Intracellular	236.1
lp_1897	6.808	pyruvate kinase	Intracellular	43.3
lp_2055	3.094	ribosomal protein S2	Intracellular	30.2
lp_2118	12.661	trigger factor; peptidylprolyl isomerase	Intracellular	49.4
lp_2417	2.485	prophage P2a protein 40; major capsid protein	Intracellular	38.1
lp_2793	3.150	Unknown	Intracellular	76.3
lp_2988	7.437	extracellular zinc metalloproteinase, M10 family (putative)	Intracellular	28.1
lp_3170	2.172	phosphoglycerate mutase family protein	Intracellular	26.1

lp_3614	462.380	Oxidoreductase	Intracellular	33.4
lp_0625	2.397	prophage P1 protein 2, mitogenic factor, cell surface lipoprotein	Lipid anchored	41.8
lp_0689	2.187	cell surface protein, lipoprotein precursor	Lipid anchored	23.1
lp_1072	43.973	thiamin biosynthesis lipoprotein ApbE	Lipid anchored	34.6
lp_1609	4.847	glycine betaine/carnitine/choline ABC transporter, substrate binding protein	Lipid anchored	53.9
lp_3686	69.772	ABC transporter, substrate binding protein	Lipid anchored	60.5
lp_0197	3.233	cell surface protein precursor	LPxTG Cell-wall anchored	105.2
lp_0800	10.941	cell surface protein precursor	LPxTG Cell-wall anchored	226.4
lp_0923	3.653	cell surface protein precursor	LPxTG Cell-wall anchored	83.3
lp_0946	2.707	mucus-binding protein (putative)	LPxTG Cell-wall anchored	37.7
lp_1643	4.321	mucus binding protein precursor	LPxTG Cell-wall anchored	22.2
lp_2925	9.621	cell surface protein precursor	LPxTG Cell-wall anchored	89.5
lp_2940	11.259	cell surface protein precursor	LPxTG Cell-wall anchored	44.3
lp_3114	8.059	mucus-binding protein (putative)	LPxTG Cell-wall anchored	214.8
lp_0802	4.387	polar amino acid ABC transporter, substrate binding and permease protein	Multi-transmembrane	52.3
lp_0856	3.466	acyltransferase (putative)	Multi-transmembrane	74.4
lp_2914	2.922	integral membrane protein	Multi-transmembrane	66.8
lp_0043	2.774	serine protease HtrA	N-terminally anchored	43.1
lp_0542	40.886	septum formation initiator	N-terminally anchored	15.2
lp_0593	2.916	transcriptional attenuator, cell envelope-related, LytR family	N-terminally anchored	43.6
lp_0600	4.196	extracellular zinc metalloproteinase, M10 family (putative)	N-terminally anchored	26.5
lp_0628	4.558	prophage P1 protein 5, superinfection exclusion (cell surface N-anchored)	N-terminally anchored	37.9
lp_0688	2.626	DNA entry nuclease	N-terminally anchored	36.2
lp_0746	20.051	phosphate ABC transporter, substrate binding protein	N-terminally anchored	31.5
lp_1568	4.064	transpeptidase (penicillin binding protein 2B)	N-terminally anchored	34.8
lp_1751	31.422	transpeptidase-transglycosylase (penicillin binding protein 1A)	N-terminally anchored	50.0
lp_2737	16.042	cell surface hydrolase, DUF915 family, membrane-bound (putative)	N-terminally anchored	29.7
lp_2809	9.599	extracellular protein (putative)	N-terminally anchored	23.2

lp_2812	4.905	extracellular protein, membrane-anchored (putative)	N-terminally anchored	28.5
lp_2845	13.153	extracellular transglycosylase (putative)	N-terminally anchored	30.7
lp_3014	3.529	extracellular transglycosylase (putative)	N-terminally anchored	21.3
lp_3093	4.562	muramidase (putative)	N-terminally anchored	89.6
lp_3411	9.962	extracellular protein, DUF1002 family	N-terminally anchored	34.2
lp_3421	3.818	extracellular protein, gamma-D-glutamate-meso-diaminopimelate muropeptidase (putative)	N-terminally anchored	36.8
lp_0092	29.929	ABC transporter, substrate binding protein, oligopeptide	Secretory(released)	59.8
lp_0141	6.321	extracellular protein	Secretory(released)	28.6
lp_0302	3.404	extracellular transglycosylase (putative)	Secretory(released)	26.3
lp_0304	5.058	extracellular transglycosylase (putative)	Secretory(released)	22.1
lp_0924	4.442	extracellular protein	Secretory(released)	68.6
lp_1697	19.569	adherence protein, chitin-binding domain	Secretory(released)	31.6
lp_2541	2.890	ABC transporter, substrate binding protein	Secretory(released)	35.0
lp_2978	2.253	extracellular protein	Secretory(released)	25.5
lp_3189	3.826	serine-type D-Ala-D-Ala carboxypeptidase	Secretory(released)	47.0
lp_3412	4.978	extracellular protein	Secretory(released)	118.7
lp_3414	3.493	extracellular protein	Secretory(released)	27.4
lp_3676	12.665	extracellular protein	Secretory(released)	56.8
lp_2960	4.815	lipase/esterase, subfamily of SGNH-hydrolases	transmembrane	25.5

[a] FDR Relative abundances were calculated by the ratio of Label-free quantitation (LFQ) of detected peptides in wild type and *lgt* mutant.
